# Monitoring of Sperm-Independent Calcium Oscillations in Immature Oocytes of Mice

**DOI:** 10.21769/BioProtoc.5576

**Published:** 2026-02-05

**Authors:** Sae Horiike, Woojin Kang, Ban Sato, Kenji Miyado, Hidehiko Ogawa

**Affiliations:** 1 Department of Bioscience, Graduate School of Life Science, Tokyo University of Agriculture, Setagaya-ku, Tokyo, Japan; 2 Laboratory Animal Resource Center, Transborder Medical Research Center, Institute of Medicine, University of Tsukuba, Tsukuba, Ibaraki, Japan; 3 Department of Life Sciences, School of Agriculture, Meiji University, Tama-ku, Kawasaki, Kanagawa, Japan; 4 Department of Reproductive Biology, National Research Institute for Child Health and Development, Setagaya-ku, Tokyo, Japan

**Keywords:** Ca^2+^ oscillations, Immature oocyte, Ovary, EDTA, Mouse

## Abstract

Repetitive increases of intracellular calcium ions (Ca^2+^ oscillations) control cellular functions in various biological events, including meiotic resumption after fertilization. Sperm-derived substances enter the cytoplasm of mature oocytes by sperm fusion, causing Ca^2+^ oscillations. Sperm-independent Ca^2+^ oscillations are also induced in immature oocytes isolated from the ovaries of neonatal to adult mice. The presence of Ca^2+^ oscillations may contribute to subsequent oocyte quality; however, its physiological role and molecular mechanism are unclear. Here, we describe a method of collecting immature oocytes from the ovaries of juvenile (12, 15, and 21 days after birth) and adult mice and monitoring their Ca^2+^ oscillations. Since mouse oocytes are larger than other types of cells, they are a useful model for studying spatiotemporal patterns and the mechanism of Ca^2+^ oscillations in various types of cells. This method can be applied to other rodents due to similarities in oocyte size and developmental processes. Furthermore, the use of various fluorescent probes enables visualization of organelle rearrangement. The mechanism of interaction between oocytes and somatic cells differs between juvenile and adult mice. Therefore, two distinct methods are employed for oocyte collection.

Key features

• Isolation of immature oocytes from juvenile ovaries [use of ethylenediaminetetraacetic acid (EDTA)].

• Isolation of immature oocytes from adult ovaries (no treatment with protease and EDTA).

• Monitoring of Ca^2+^ oscillations in immature oocytes.

## Graphical overview


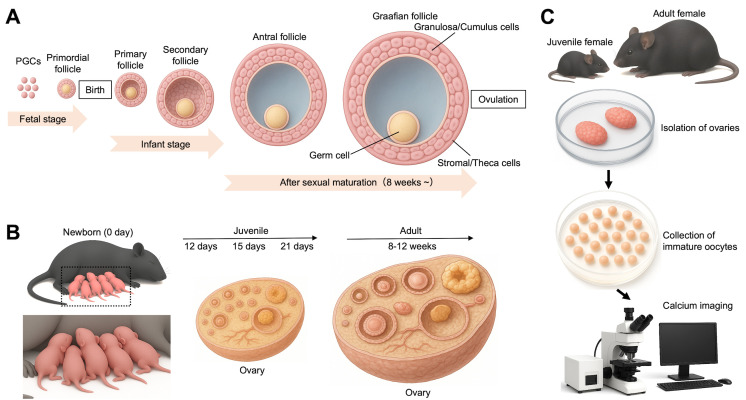


**Monitoring of Ca^2+^ oscillations in immature oocytes from juvenile and adult mice.** (A) An overview of follicular development from fetal to adult stages. Primordial germ cells (PGCs) are precursors of germline cells, including oocytes within ovarian follicles. In mammalian females, ovarian follicles form during the fetal or early neonatal period, and the pool of oocytes is considered finite at birth. (B) Representation of mice used in this protocol. After newborn mice (0 day) are raised, 12-, 15-, and 21-day-old and 8–12-week-old female mice are used here. (C) Monitoring of Ca^2+^ oscillations. Immature oocytes are collected from the ovaries of juvenile and adult female mice.

## Background

Oscillations in the concentration of intracellular calcium ions (Ca^2+^ oscillations) occur in various types of cells. Ca^2+^ oscillations control an array of cellular processes, including cell metabolism, exocytosis, and cell cycle progression [1]. Ca^2+^ oscillations arise from integrated actions of multiple organelles. Mainly, the endoplasmic reticulum (ER) and mitochondria work as drivers and modulators [2]. The plasma membrane maintains the supply of Ca^2+^ and resets Ca^2+^ oscillations [3]. The nucleus interprets oscillatory signals to control long-term cellular responses [4]. These organelles cooperatively regulate Ca^2+^ oscillations in various types of cells, such as neural cells [5], muscular cells [6], immune cells [7], and oocytes [1]. Immediately after sperm fusion during fertilization, sperm-derived substances (sperm factors) enter the cytoplasm of mature oocytes and evoke Ca^2+^ oscillations, subsequently leading to meiotic resumption. Sperm-derived phospholipase Cζ1 (PLCZ1) is an enzyme that induces Ca^2+^ oscillations in mammalian mature oocytes. Moreover, extramitochondrial citrate synthase triggers an initial spike of Ca^2+^ oscillations in the presence of PLCZ1, implying that these two sperm factors work independently at least in mice. On the other hand, Ca^2+^ oscillations are spontaneously induced in various stages of immature oocytes [1]. Both fertilization and oocyte maturation are prerequisites for embryonic development. However, the role of Ca^2+^ oscillations in immature oocytes is still unclear, although preventing intracellular Ca^2+^ changes inhibits meiotic maturation at specific stages of oocytes [8]. Studies on how Ca^2+^ oscillations regulate oocyte maturation may provide new insights into the causes of female infertility. Moreover, mouse oocytes are a useful model to study the spatiotemporal mechanism of Ca^2+^ oscillations. This method is likely applicable to other rodents with structurally similar ovaries. Observation of calcium oscillations in oocytes is most often performed on oocytes after fusion with sperm. This method is useful for efficiently collecting oocytes within the ovary.

## Materials and reagents

**Biological materials**

1. C57BL/6J female mice (Japan SLC, Inc.): 12, 15, and 21 days and 8–12 weeks after birth

**Reagents**

1. M2 medium (Sigma-Aldrich, catalog number: MR-015), store at 4 °C

2. L-15 medium (WAKO, catalog number: 128-06075), store at 4 °C

3. Liquid paraffin (Nacalai Tesque, catalog number: 26137-85, specially prepared reagent), store at room temperature (RT) and in the dark, away from sunlight

4. 70% ethanol (Yoshida Pharmaceutical Company, Ecosyoueta disinfectant solution, catalog number: 14987288980046)

5. UltraPure^TM^ 0.5 M ethylenediaminetetraacetic acid (EDTA), pH 8.0 (Invitrogen, catalog number: 15575020), store at RT

6. Sodium chloride (NaCl) (Nacalai Tesque, catalog number: 31320-05), store at RT

7. Potassium chloride (KCl) (Nacalai Tesque, catalog number: 28514-75), store at RT

8. Calcium chloride (CaCl_2_) (Nacalai Tesque, catalog number: 06729-55), store at RT

9. Potassium dihydrogen phosphate (KH_2_PO_4_) (Nacalai Tesque, catalog number: 28721-55), store at RT

10. Magnesium sulfate heptahydrate (MgSO_4_·7H_2_O) (Nacalai Tesque, catalog number: 21003-75), store at RT

11. Sodium hydrogen carbonate (NaHCO_3_) (Nacalai Tesque, catalog number: 31213-15), store at RT

12. D-(+)-Glucose (Nacalai Tesque, catalog number: 168-06), store at RT

13. Pyruvic acid sodium salt (Nacalai Tesque, catalog number: 29806-12), store at 4 °C

14. Penicillin G potassium salt (Sigma-Aldrich, catalog number: 113-98-4), store at 4 °C

15. Streptomycin sulfate salt (Sigma-Aldrich, catalog number: S6501-25G), store at 4 °C

16: Albumin, from bovine serum, Cohn fraction V, pH 7.0 (albumin fraction V) (WAKO, catalog number: 017-23294), store at 4 °C

17. Oregon green 488 BAPTA-1 AM (Invitrogen, catalog number: O6807), store at 4 °C

18. UltraPure^TM^ 0.5 M EDTA, pH 8.0 (ThermoFisher, catalog number: 15575020), store at RT

19. Dulbecco's phosphate buffered saline (Nacalai Tesque: 14249-24), store at RT

**Solutions**

1. Toyoda-Yokoyama-Hoshi (TYH) medium (see Recipes)

2. EDTA-containing PBS(-) (see Recipes)

**Recipes**

**1. TYH medium**

**Table:** 

Reagent	Final concentration	Quantity or volume
NaCl	-	697.6 mg
KCl	-	35.6 mg
CaCl_2_	-	19.0 mg
KH_2_PO_4_	-	16.2 mg
MgSO_4_·7H_2_O	-	29.3 mg
NaHCO_3_	-	210.6 mg
D-(+)-Glucose	-	100.0 mg
Pyruvic acid sodium salt	-	5.5 mg
Penicillin G potassium salt	-	7.5 mg
Streptomycin sulfate salt	-	5.0 mg
Albumin fraction V	-	400.0 mg
Distilled H_2_O	-	Up to 100 mL

**2. EDTA-containing PBS(-)**

**Table:** 

Reagent	Final concentration	Quantity or volume
UltraPure^TM^ 0.5 M EDTA, pH 8.0	0.1 mM	-
Dulbecco's phosphate buffered saline	-	Up to 1 mL

**Laboratory supplies**

1. Glass pipettes (Drummond Scientific Company, MICROCAPS^®^, catalog number: 1-000-0500)

2. 1.5 mL microcentrifuge tubes (WATSON, catalog number: 131-815C)

3. 50 mL tubes (Greiner, catalog number: 227261)

4. 10 mL plastic pipettes (FALCON, catalog number: 357551)

5. 1,000 μL pipette tips (WATSON, catalog number: 110-7-6C)

6. 200 μL pipette tips (WATSON, catalog number: 110-705C)

7. 10 μL pipette tips (WATSON, catalog number: 110-207C)

8. Kimwipes (NIPPON PAPER CRECIA, catalog number: 62020)

9. Aluminum foil (Mitsubishi aluminum, catalog number: B0093XFMQC)

10. Paper towels (ASKUL, catalog number: 1944368)

11. 35 mm dishes (IWAKI, catalog number: 1000-035)

12. 60 mm dishes (CORNING, catalog number: 351007)

13. Syringes with needles (Terumo Corporation, 1 mL syringe with 26-gauge 1/2-inch needle, catalog number: SS-01T2613S)

14. Disposable gloves (AXEL, catalog number: 61-7347-30)

15. Protective equipment (e.g., masks, goggles, and lab coats)

## Equipment

1. CO_2_ incubator (WAKENYAKU, model: 9300E)

2. Stereomicroscope (Nikon, model: SMZ645)

3. Hot plate (NISSIN, model: NHP-M20)

4. Electronic balance (Mettler Toledo, model: Newclassic MS)

5. Gas torch (PRINCE, model: GB-2001)

6. Pipette controller (Drummond, model: Pipet-Aid XP)

7. P-1000 pipette (Gilson, model: F120602)

8. P-200 pipette (Gilson, model: F123601)

9. Micropipette (Eppendorf, model: 4920000024)

10. Mouth pipette (Drummond, model: 2-040-000)

11. Ampoule glass cutter (AXEL, model: 5-124-22)

12. Large straight scissors (Natsume Seisakusho, model: B-3)

13. Small straight scissors (Natsume Seisakusho, model: B-12)

14. Tweezers (AXEL, model: 2-529-12)

15. Precision tweezers (DUMONT, model: NO.5-INOX)

16.18-gauge needle (TERUMO, model: NN-1838R)

17. 21-gauge needle (TERUMO, model: NN-2138S)

18. 5-mL syringe (TERUMO, SS-05SZ)

19. 1-mL syringe (TERUMO, SS-01T)

20. Dispenser for liquid paraffin (Nichiryo Co., Ltd., model: 00-DP-2B)

21. Plastic cages (Clea Japan, model: CL-0103-2 Mouse TPX)

22. Confocal microscope system (Yokogawa Electric, model: CSU-Frontier-SESP1)

23. Stage top incubator (Tokai Hit, INUBG2-PPZI)

24. Highly sensitive CCD camera (Andor Technology, model: iXon EMCCD)

## Software and datasets

1. R software (version 4.2.2) (https://www.r-project.org/)

2. Imaging software Andor iQ (Andor Technology: version 1.9.1)

## Procedure

**A. Animals**

1. Breed mice (2–4 mice per cage) under the following specific pathogen-free conditions: 23 ± 1 °C, 12 h light/dark cycles (light on at 8:00 and off at 20:00), and ad libitum access to food and water.

**B. Preparation of glass pipettes for oocyte handling**

**Figure 1.**
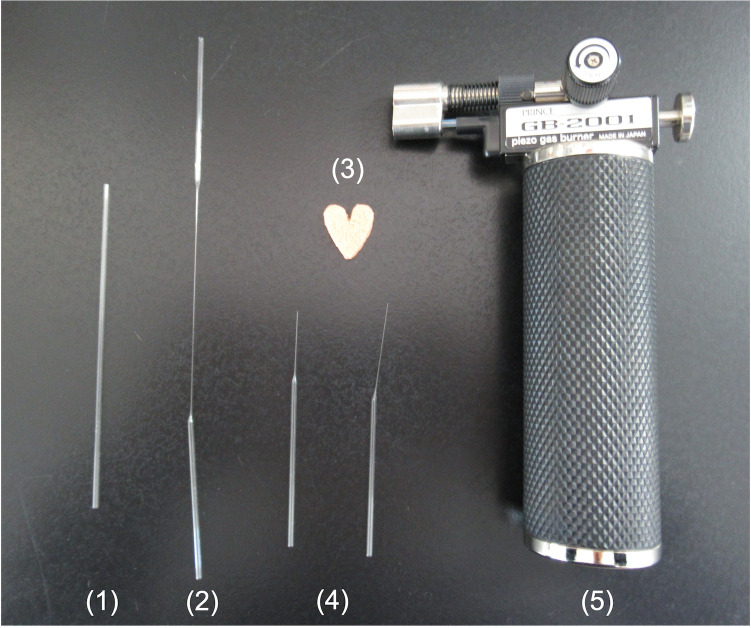
Preparation of glass pipettes for oocyte handling. 1. Glass pipette. 2. Extended glass pipette. 3. Ampoule glass cutter. 4. Cut glass pipette. 5. Gas burner.

1. Cut the glass pipettes in the middle using an ampoule glass cutter (Figure 1).

2. Heat the cut edge of glass pipettes with the flame of an alcohol lamp to smooth.

3. Hold both sides of the glass pipette with one hand and a pair of curved tip tweezers.

4. Heat the middle of the pipette until it is softened slightly, remove from the flame, and immediately pull both ends horizontally (Figure 1).

5. Cut the excess part of the pipette, heat, and smooth the tip slightly. The smooth tip is less likely to damage the oocytes and dishes (Figure 1).

6. Check the tip under a stereomicroscope and choose glass pipettes with an inner diameter of approximately 150–200 μm.

7. Wrap the glass pipettes with aluminum foil, sterilize by dry-heat (180 °C, 30 min), and store in a new 15 mL tube.

**C. Preparation of dishes**

1. Place TYH medium drops (100 μL per drop) in 60 mm dishes and cover these drops with liquid paraffin.

2. Incubate these dishes in a CO_2_ incubator at 37 °C with 5% CO_2_ for at least 60 min before collecting immature oocytes.

3. Thaw M2 medium and place four drops (100 μL per drop) in a new 60 mm dish.

*Note: Prepare this just before washing the oocytes. There is no need to cover M2 medium drops with liquid paraffin and to place them in the CO_2_ incubator.*

**D. Collection of immature oocytes from juvenile mice**

**Figure 2.**
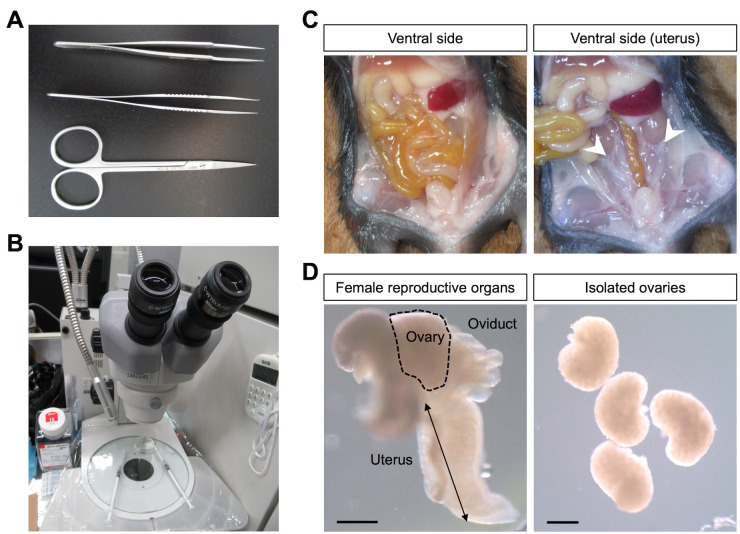
Isolation of ovaries from juvenile mice. (A) Anatomical set for abdominal exploration. (B) A stereomicroscope. (C) View after an abdominal operation (left image) and after turning over an intestine (right image) in female mice (12 days after birth). Arrowheads indicate ovaries. (D) Microscopic images of the oviduct and the uterus (left image) and isolated ovaries (right image). Scale bars, 1 mm.

**Figure 3.**
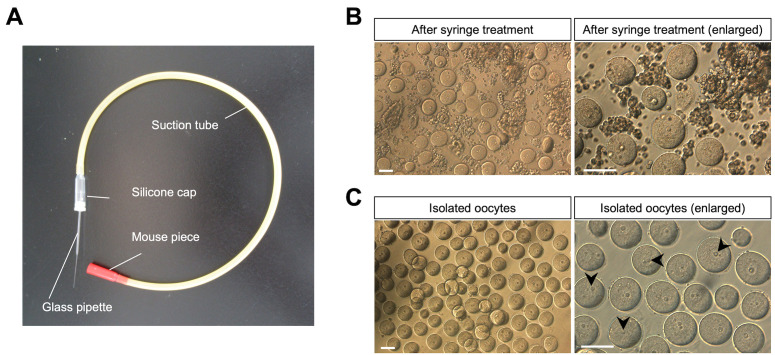
Collection of immature oocytes from juvenile mice and monitoring of Ca^2+^ oscillations. (A) A mouth pipetting set for oocyte transfer. (B) Solution of oocytes and somatic cells after syringe treatment. Ovaries are isolated from female mice (12 days after birth). After the fragmented ovaries are treated with the syringe, oocytes and somatic cells are observed in the M2 solution (upper images). (C) Oocyte retrieval. The oocytes are collected using the mouse pipetting set. Arrowheads represent oocyte nuclei. Scale bars, 50 μm.

1. Place scissors and tweezers on the bench and wear gloves (and other protective equipment as necessary) (Figure 2A, B).

2. At 12, 15, and 21 days after birth, sacrifice female mice using carbon dioxide.

3. Disinfect the abdomen with 70% ethanol and cut the abdominal skin of mice with a midline incision from the abdomen to the chest using large scissors.

4. Pull two sides of the cut skin and access the mouse peritoneum.

5. Cut the peritoneum using small scissors (Figure 2C).

6. Locate the V-shaped uterus, oviducts, and ovaries by shifting the position of the gut and internal organs (Figure 2C).

7. Hold the utero-tubal junction with a pair of precision tweezers and separate the ovary from the oviduct and the uterus (Figure 2D).

8. Clean the ovary by removing adipose tissues and transfer the ovary to the dish from step C2 (Figure 2D).

9. Using a 22-G needle, dissect each ovary into tissue fragments in M2 medium (Figure 3A, B).

10. Centrifuge at 1,000× *g* for 1 min at 24 °C.

11. Remove the supernatant.

12. Add 400 μL of L-15 medium containing 0.1% collagenase and incubate at 37 °C for 10 min.

13. Perform tapping and then centrifuge at 1,000× *g* for 1 min at 24 °C.

14. Remove the supernatant.

15. Add 200 μL of EDTA-containing PBS(-) and incubate at 37 °C for 5 min.

16. Perform tapping and then centrifuge at 1,000× *g* for 1 min at 24 °C.

17. Remove the supernatant.

18. After suspending for 10 min in M2 medium, collect oocytes visually under a stereomicroscope using a capillary (Figure 3C).

**E. Collection of immature oocytes from adult mice**

1. Follow steps D1–11.

2. After suspending for 10 min in M2 medium, collect oocytes visually under a stereomicroscope using a capillary.

*Notes:*

*1. Oocytes are larger than somatic cells, measuring 50 μm in diameter, and are spherical with a nucleus at their center. This distinctive shape makes them easily distinguishable from other cells. Furthermore, they are surrounded by the zona pellucida, making them resistant to physical stress. To prevent damage to the oocytes, it is important to select a glass capillary with a diameter slightly larger than the oocyte itself.*

*2. The bond between the oocyte and somatic cells is weak, allowing the oocyte to be released from ovarian fragments under syringe pressure. However, repeated syringe manipulation increases the risk of damaging the oocytes. Three manipulations with the syringe are optimal.*

**F. Monitoring of Ca^2+^ oscillations**

**Figure 4.**
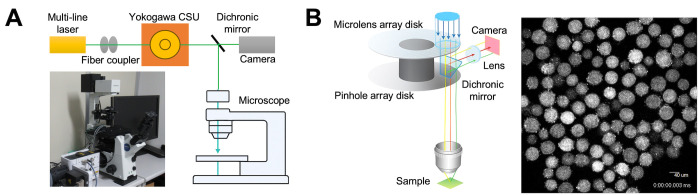
Schematic image of time-lapse analysis. (A) Confocal microscope system (CSU-Frontier-SESPI). This system includes a microscope, CSU confocal scanner unit, and incubator. (B) Time-lapse acquisition. The CSU confocal scanner unit produces data suitable for the quantitative analysis of large datasets. Images captured in Z-stacks can be easily reconstructed into 3D models for analysis. A captured image is shown on the right.

1. Incubate immature oocytes (around 100 oocytes) in TYH medium containing the Ca^2+^-sensitive fluorescent dye Oregon green 488 BAPTA-1 AM (final concentration of 2 μM) for 15 min at 37 °C in a CO_2_ incubator.

2. Wash immature oocytes three times with TYH medium (5 min each).

3. Use a stage top incubator to control temperature, humidity, and CO_2_ concentration (5%) for live cell imaging (Figure 4A).

4. Capture fluorescent images of the oocytes using a highly sensitive CCD camera, using the specific software to operate the camera, every 10 s (Figure 4B).

*Note: In our work, the magnification of the Olympus UIS2 UPLANFL objective lens was 10×, and a pinhole diameter (25 μm) was used for low magnification and a wider field of view. Scanning speed was capable of high-speed imaging up to 2,000 frames per second. Wavelength range was compatible with standard laser excitation lines from 405 to 640 nm.*

**Figure 5.**
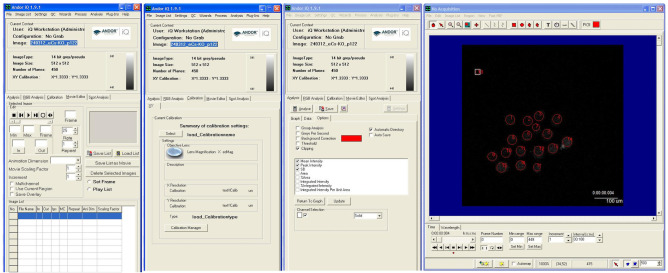
Time-lapse processing and data analysis. The data is processed using analysis software (Andor iQ). The red circles indicate oocytes. A single analysis can quantify the fluorescence intensity of multiple oocytes. The image exhibits three modes (movie editor, calibration, and analysis).

5. Measure fluorescence intensity by using the imaging software Andor iQ. Subtract each fluorescent image (F) from the image before injection or the image with the lowest fluorescence intensity (F0). Keep all acquisition parameters constant within each experiment to allow quantitative comparison between oocytes (Figure 5).

6. Measure the fluorescence intensity for individual oocytes within a user-selected region that covers the majority of the area of each oocyte.

7. The mean intensity over the same area for each of the images in a time series is analyzed automatically.

8. Report changes in fluorescence intensity as the F/F0 ratio.

*Note: Exposure time was optimized to maximize signal intensity while avoiding pixel saturation. Typical exposure times ranged from 50 to 300 ms, depending on probe brightness and labeling efficiency. Saturation levels were monitored using the Andor iQ intensity histogram, and acquisition settings were adjusted to ensure that the dynamic range of the camera was fully utilized without clipping. Excitation light intensity was minimized to reduce photobleaching and phototoxicity, particularly important for large and optically sensitive mouse oocytes. Z-stack parameters were defined to encompass the entire oocyte volume. Optical sections were acquired at intervals of 1 μm, depending on the objective numerical aperture and axial resolution. Maximum intensity projections or single optical sections were generated post-acquisition using Andor iQ analysis tools.*

## Data analysis

**Figure 6.**
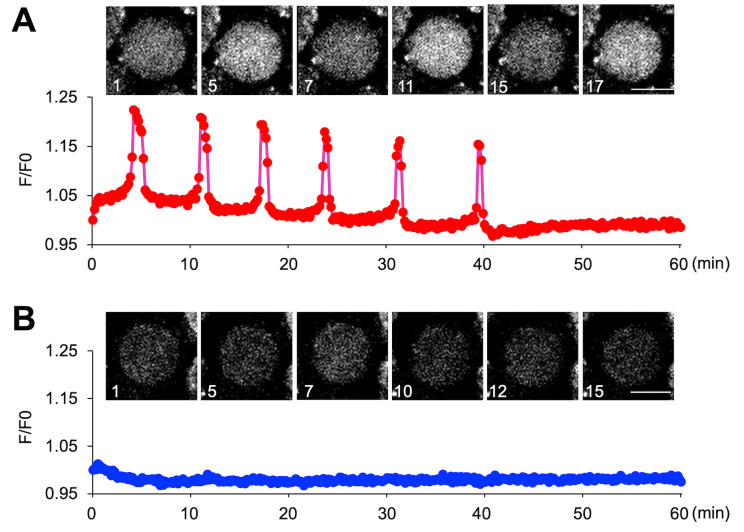
Monitoring Ca^2+^ oscillations in immature oocytes. (A) Ca^2+^ oscillation-positive oocytes. (B) Ca^2+^ oscillation-negative oocytes. For the oocytes observed in Video 1, graphs were created for an oocyte that exhibited calcium oscillations and an oocyte that did not.

1. Analyze the data using imaging software Andor iQ (version 1.9.1) and R software (version 4.2.2) (Figure 5).

2. Images can be exported from Andor iQ using any of the following formats: AVI, BioradMRC, Bitmaps, Fenestra, JPEG, Kontron, Micro Voxel, Photometrics, and TIFF.

3. The *Analysis* tab is used to calculate statistics for the current image or, if drawn onto the image, user-defined regions. Results are displayed in both graphical and numerical formats.

4. The *Spot Analysis* tool is used to quantify the morphology, density, and intensity of discrete objects.

5. Raw data is output as text files.

6. Graphs can be generated using R software. Data are imported as data frames, and figures are created primarily using the ggplot2 package. Appropriate geometric layers are applied depending on the data type, with axes labels, legends, and themes customized for clarity. Final figures can be exported in high-resolution formats suitable for publication (Figure 6 and Video 1).

**Video 1.**
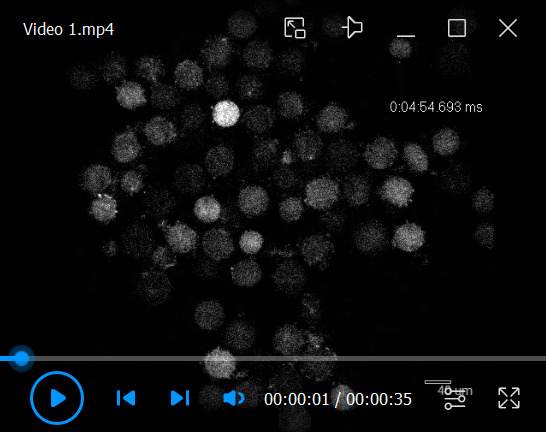
Monitoring Ca^2+^ oscillations of immature oocytes. The oocytes were collected from female mice at 12 days after birth. Approximately 200 oocytes were collected from the ovaries of three mice. An example of the parameters is indicated in Figure 5.

## Validation of protocol

This protocol has been used and validated in the following research article:

• Horiike et al. [9]. Biological significance of sperm-independent calcium oscillations in immature oocytes of mice. *MicroPubl Biol* 2025, 2025. https://doi.org/10.17912/micropub.biology.001748

## General notes and troubleshooting

**General notes**

1. Producing mouse capillaries requires skill and expertise.

2. After fragmenting the ovaries of juvenile mice, processing them with protease alone without EDTA results in high viscosity, making oocyte recovery difficult. Since the viscosity is too high to retrieve the oocytes, the collection of new ovaries is needed.

3. The bond between the oocyte and somatic cells is weak, allowing the oocyte to be released from ovarian fragments under syringe pressure. Especially when recovering oocytes from the ovaries of mature mice, moderate pressure applied with a syringe allows for oocyte retrieval without protease and EDTA treatment. Concretely, repeated syringe manipulation increases the risk of damaging the oocytes. Three manipulations with the syringe are optimal.

**Troubleshooting**

**Problem 1:** When collecting oocytes from the ovaries of mature mice, oocytes may not be retrieved even after repeatedly pushing and pulling ovarian fragments with a syringe. In such cases, reducing the syringe gauge size may enable successful recovery.

**Problem 2:** When collecting oocytes from the ovaries of mature mice, increasing the number of times ovarian fragments are aspirated with a syringe does not result in additional oocyte recovery. It may actually break the oocytes.
